# Health Care Consumer Shopping Behaviors and Sentiment: Qualitative Study

**DOI:** 10.2196/13924

**Published:** 2020-06-16

**Authors:** Deborah Gordon, Anna Ford, Natalie Triedman, Kamber Hart, Roy Perlis

**Affiliations:** 1 Mossavar-Rahmani Center for Business and Government Harvard Kennedy School Cambridge, MA United States; 2 Center for Quantitative Health Massachusetts General Hospital Boston, MA United States

**Keywords:** decision making, choice behavior, mental processes, behavioral economics, health costs, health care costs, treatment costs, cost sharing, health expenditures, out-of-pocket costs

## Abstract

**Background:**

Although some health care market reforms seek to better engage consumers in purchasing health care services, health consumer behavior remains poorly understood.

**Objective:**

This study aimed to characterize the behaviors and sentiment of consumers who attempt to shop for health care services.

**Methods:**

We used a semistructured interview guide based on grounded theory and standard qualitative research methods to examine components of a typical shopping process in a sample size of 54 insured adults. All interviews were systematically coded to capture consumer behaviors, barriers to shopping behavior, and sentiments associated with these experiences.

**Results:**

Participants most commonly described determining and evaluating options, seeking value, and assessing or evaluating value. In total, 83% (45/54) of participants described engaging in negotiations regarding health care purchasing. The degree of positive sentiment expressed in the interview was positively correlated with identifying and determining the health plan, provider, or treatment options; making the decision to purchase; and evaluating the decision to purchase. Conversely, negative sentiment was correlated with seeking value and making the decision to buy.

**Conclusions:**

Consumer shopping behaviors are prevalent in health care purchasing and can be mapped to established consumer behavior models.

## Introduction

To mitigate rising health costs [1], employers and health insurers have increased consumer cost sharing in health insurance plan design [2,3]. Such consumer-driven health care, where consumers shoulder a greater portion of health expenditures, aims to drive down health spending by discouraging unnecessary utilization and encouraging comparison shopping for the best value care [4,5]. Despite shifting incentives [6] and related cost savings [7,8], not all savings reflect the avoidance of unnecessary or higher-priced care [9]. Recent studies have shown that simply shifting costs to consumers does not yield expected shopping behaviors. Rather, consumers often avoid necessary or preventive care [10,11]. Studies also show that consumers rarely compare prices even when tools are available [12-14], suggesting that consumer-driven health care—commonly defined narrowly by the presence of high deductibles—does not promote health care shopping.

The increased focus on consumer experience has led to investigations of consumer sentiment regarding health care encounters [15-17]. These studies highlight the applicability of automated techniques for coarsely analyzing consumer sentiment in health care and make such an analysis of large-scale unstructured data possible. However, such studies do not evaluate the association between such a sentiment and specific aspects of health care decision-making or shopping processes.

The obstacles to shopping for health care coverage and services are well documented. Information asymmetry, complexity, and patient-provider power dynamics are just a few barriers to consumer shopping [18-20]. The lack of transparency of prices—a particularly concrete and potentially addressable obstacle facing consumers—has scarcely improved in recent years despite legislative requirements and concerted efforts [21-23]. Beyond the availability and adoption of price comparison tools—and a relatively narrow definition of shopping as simply comparing prices—little is understood about how consumers could be more effectively engaged in health care shopping.

Numerous models of consumer purchasing behavior exist [24,25], but less is known about how these models apply in health care contexts. Evidence of consumer interest in engaging in health care purchasing [26] suggests opportunities for consumer-driven health care to fulfill its promise, yet also highlights gaps between consumer intention and behavior. To identify these opportunities and better explain these gaps, we sought to understand individual health care purchasing processes through a consumer lens. A deeper understanding of consumers’ health care purchasing experiences would enable health care organizations and policy makers to design interventions to efficiently engage consumers and help improve consumer value in the US health care market. Specifically, understanding the aspects of shopping that consumers find particularly challenging, or gratifying, should aid in the development of interventions to facilitate such processes.

## Methods

### Overview

We recruited a convenience sample (N=54) of individuals aged 18 to 98 years based on a study protocol and obtained a consent form approved by the human subjects review committee of the Harvard Kennedy School. To preserve participant confidentiality, considering reidentifiability, consent forms do not provide for release of individual data. Intensive interviews [26] were conducted by phone or in person by the senior investigator, an experienced qualitative interviewer, and 2 Masters-level student researchers trained by the senior investigator. All but 5 interviews were recorded and professionally transcribed; where participants did not consent to recording or recording was not available, researchers captured participant responses in detailed interview notes.

Interviewers categorized each participant by insurance type, age, and gender based on self-reports. Similarly, participants were categorized by health-related characteristics including health status and utilization, either explicitly articulated by participants or inferred by the researchers. In cases where the participant was not explicit and it was not clear from context, we categorized participants as “not reported” for that measure.

**Figure 1:**
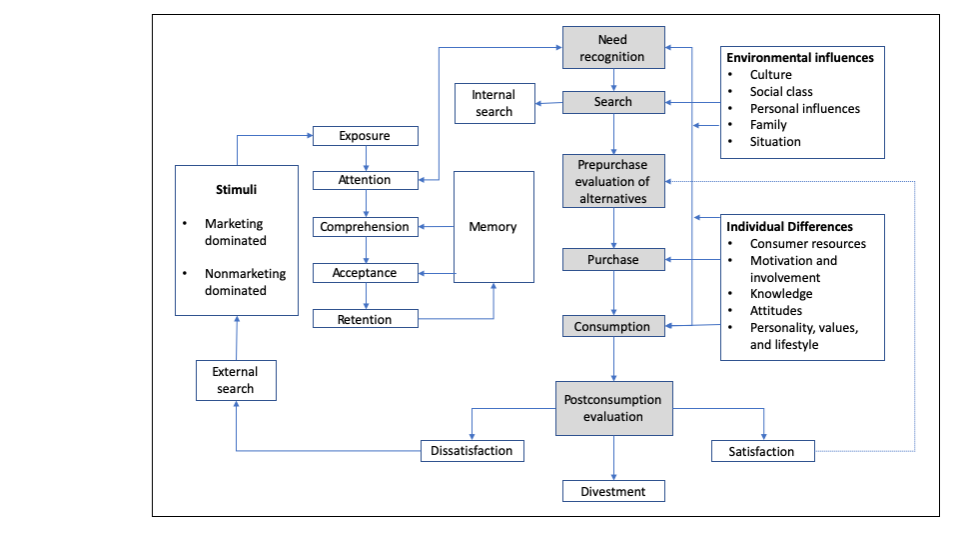
Consumer behavior model.

Interviewers used a semistructured interview guide developed by the research team, and based on grounded theory, a systematic empirical research methodology was used to construct the theory inductively via methodical data gathering and analysis [27,28]. Interviews lasted approximately one hour and were organized around components of a typical shopping process, such as the consumer decision process depicted in Blackwell/Miniard’s model (Figure 1) [25], adapted to include the following:

1. Identifying the need or desire for a health care purchase (need recognition)
2. Determining and evaluating options to meet that need or desire (search/prepurchase evaluation of alternatives)
3. Making the decision to purchase (purchase/consumption)
4. Evaluating the decision to purchase (postconsumption evaluation/divestment)

Within this shopping framework, we examined behaviors relevant to consumer value capture, such as trying to understand costs before seeking care or negotiating the cost of care before or after a service, advocating for one’s self, or making trade-offs such as paying more for convenience or accepting low-quality service to save money.

We also sought to identify barriers to traditional consumer behaviors and value capture. Barriers were either systemic (eg, administrative hassles or lack of price transparency) or consumer limitations that constrained their ability to capture value (eg, ignorance or confusion about how to capture value).

Finally, we sought to capture participant sentiment. Positive sentiments included feelings such as gratitude, relief, peace of mind, or optimism. Negative sentiments included anger, frustration, despair, anxiety, or pessimism.

### Data Analysis

Transcripts and interview notes were processed using Dedoose (SocioCultural Research Consultants, LLC), an application for managing, analyzing, and presenting qualitative and mixed methods research data [29]. The authors developed an initial code set organized around the typical shopping process components investigated in the interview guide. Following grounded theory methods [26], additional codes were created to capture emergent themes such as sentiments about shopping and beliefs about the health care system. Researchers coded each transcript, and the senior investigator reviewed all codes in all transcripts to ensure consistency in the application of codes.

For the examination of sentiment associated with behavior, specific codes were identified to indicate either positive or negative feelings. Positive sentiment included the codes for optimism bias, peace of mind/comfort, and gratitude/relief. Negative sentiment included the codes for pessimism bias, vulnerability, anger/frustration, despair/desperation, fear/anxiety, and financial anxiety/concerns about cost.

As this was intended primarily as a hypothesis-generating study, the majority of analyses were descriptive, examining the frequency with which participants described engaging in particular behaviors or encountering particular scenarios. We calculated power to detect a given theme as described by Fugard and Potts [30]; for a theme with 10% population prevalence, power exceeds 90% to detect that theme at least three times in an interview cohort of this size. 

The authors also examined the extent to which expressions of positive or negative consumer sentiment were associated with the discussion of each shopping process stage. For these tests, Pearson correlations were utilized to examine the relationship between the frequency of code pairs, with a sensitivity analysis using linear regression to adjust for effects of age and gender. All analyses utilized Stata SE 13.1 [31]; figures were generated using R version 3.5.0 [32]. While nominal *P* values are reported, for secondary analyses, we focused on the magnitude of effect (eg, correlation).

## Results

### Participant Details

A total of 54 interviews were completed with individual consumers. All participants were currently insured; 65% (35/54) were women, and the mean age was 43 (SD 16.23) years (Table 1). Despite the majority (49/54, 91%) reporting being in good-to-excellent health, health care utilization was estimated as moderate or high for 80% (43/50) of participants. More than half the participants (30/54, 56%) reported either a chronic condition or a past catastrophic accident or emergency requiring follow-up care. A minority (5/54, 9% of all participants) reported a current or previous cancer diagnosis.

Participant responses revealed that in addition to 4 basic stages in a shopping process, seeking value before a purchase and assessing value following a purchase were important components in the health care purchase processes. Thus, the analysis incorporated these 2 concepts:

- Stage 1: Identifying the need or desire for a purchase in a health care context
- Stage 2: Determining and evaluating options to meet that need or desire
- Stage 3: Seeking value (a subset of determining/evaluating options)
- Stage 4: Making the purchase decision
- Stage 5: Evaluating the purchase decision in terms of quality and/or satisfaction
- Stage 6: Assessing value (a subset of evaluating the purchase decision)

The distribution of participant responses by shopping process stage is reported in Multimedia Appendix 1. Nearly every participant addressed each shopping stage at least once; the shopping stage discussed most often was Stage 2 (determining and evaluating options), followed by Stage 3 (seeking value), and Stage 6 (assessing value). Participants with individual insurance (ie, purchased on a state or federal health insurance marketplace) more frequently mentioned Stage 2 (determining and evaluating options; *t_52_*=2.90; *P*=.007).

We also categorized the most commonly cited consumer shopping behaviors, summarized in Table 2; intensity, measured by the average number of mentions per participant, is depicted in Table 3. Almost all participants (53/54, 98%) had experience paying out-of-pocket or sharing in health care or coverage costs; these experiences were discussed more than 5 times per interview, on average.

**Table 1:** Participant characteristics (N=54).

Characteristics		Values
Age (years), mean (SD)		43.44 (16.23)
**Gender, n (%)**		
	Female	35 (65)
	Male	18 (33)
	Intersex	1 (2)
**Insurance type, n (%)**		
	Employer-sponsored	24 (44)
	Individual	12 (22)
	Medicare	5 (9)
	Other insurance^a^	13 (24)
**Health status, n (%)**		
	Excellent	12 (22)
	OK/pretty good	37 (69)
	Not great	3 (6)
	Not reported	2 (4)
**Utilization, n (%)**		
	Low	11 (33)
	Moderate	26 (2)
	High	35 (65)
**Presence of a chronic condition or prior accident/emergency, n (%)**		
	Yes	30 (56)
	No	14 (26)
	Not reported	10 (19)

^a^Other insurance includes student insurance or Medicaid.

Most participants (53/54, 98%) discussed seeking or comparing price information or going to a provider where such information was explicit. Seeking cost information before a service was reported by 72% (39/54) of participants and comparing provider prices was reported by 56% (30/54) of participants.

All participants reported seeking value in some way, by responding to financial incentives, using health savings vehicles, or pursuing workarounds to capture the economic value. Seeking value had the highest number of mentions on average (6.48 per interview). Most participants (45/54, 83%) described some form of negotiating—one type of value-seeking behavior—which could include bargaining with a provider (23/54, 43%), arguing about a medical bill after a service (17/54, 31%), or negotiating with an insurance company for the approval of requested coverage (17/54, 31%). Participants who discussed negotiating with a provider referenced dentists (15/54, 27%), psychotherapists (10/54, 18%), out-of-network providers not covered by their insurance plans (10/54, 18%), hospitals or other providers with whom the participant had an outstanding balance (8/54, 15%), and other types of providers (24/54, 45%).

Though cost was a consideration for 85% (46/54) of participants, two-thirds (36/54, 67%) also discussed situations in which they were price insensitive or where factors other than price drove their care or coverage decisions. Significant positive correlation between price insensitivity in provider selection and price insensitivity in health plan selection was observed (*r*=0.32; *P*<.02). An adjustment for participant age and gender in regression models did not meaningfully change this association. Individual insurance was associated with discussing factoring costs (*t_52_*=3.30; *P*=.002), whereas employer-sponsored insurance was associated with price insensitivity (*t_52_*=−2.8; *P*=.008). Brand was not a dominant factor in participant selection of provider or health plan, arising in just 22% of interviews. 

The majority of participants had experienced systemic barriers—billing errors or insurance policies blocking needed services (Table 4)—to capturing value. All participants expressed personal barriers such as their own ignorance or attitude. Notably, 91% (49/54) of participants articulated lack of trust—reflected in questioning their provider’s authority or the motivations of their provider or health plan. Table 4 includes the frequency of each type of barrier cited.

**Table 2:** Frequency of explicit consumer shopping behaviors.

Consumer behavior		Participants (N=54), n (%)	Mentions, mean (SD)
Paying for care		53 (98)	5.07 (3.53)
**Seeking/comparing/knowing prices**		53 (98)	5.22 (3.88)
	Seeking cost/price before getting care	39 (72)	1.78 (2.00)
	Comparing prices/shopping around for better price	30 (56)	1.17 (1.49)
	Knowing costs/seeing provider with flat fees	21 (39)	0.62 (1.13)
**Negotiating/arguing bills**		45 (83)	3.26 (3.06)
	Negotiating with a provider^a^	23 (43)	0.98 (1.58)
	Arguing a bill after a service	17 (31)	0.67 (1.67)
	Negotiating with the insurance company for approval of a requested service or drug	17 (31)	0.80 (1.38)
Exhibiting self-advocacy/empowerment		48 (89)	4.52 (3.76)
Seeking value (eg, responding to incentives, using health savings)		54 (100)	6.48 (4.39)
Making trade-offs in decision making		46 (85)	2.94 (2.37)
Factoring brand in provider or plan selection		12 (22)	0.44 (1.02)
**Factoring cost in plan/provider/treatment selection**		46 (85)	4.26 (3.86)
	Factoring cost in health plan selection	28 (52)	1.22 (1.60)
	Factoring cost in provider selection	15 (28)	0.43 (0.81)
	Factoring cost in treatment decision	37 (69)	2.61 (3.37)
Price insensitivity		36 (67)	1.72 (1.78)

^a^Of the participants who discussed negotiating with providers, 27% (6/23) discussed negotiating with dentists, 18% (4/23) with psychotherapists, 18% (4/23) with out-of-network providers, 14% (3/23) with hospitals or other providers with whom the participant had an outstanding balance, 9% (2/23) discussed negotiating for medications or with pharmacies, and 5% (1/23) each with an optometrist, with a chiropractor, in regular doctor visits, and in medical tests.

**Table 3:** The intensity of discussion of consumer shopping behaviors.

Consumer behavior	Participants, n (%)	Mentions, mean (SD)
Paying for care	53 (98)	5.07 (3.53)
Seeking/comparing/knowing prices	53 (98)	5.22 (3.88)
Negotiating/arguing bills	45 (83)	3.33 (3.08)
Exhibiting self-advocacy/empowerment	48 (89)	4.52 (3.76)
Seeking value (eg, responding to incentives, using health savings)	54 (100)	6.48 (4.39)
Making trade-offs in decision-making	46 (85)	2.94 (2.37)
Factoring brand in provider or plan selection	12 (22)	0.44 (1.02)
Factoring cost in provider/plan/treatment selection	46 (85)	4.26 (3.86)
Price insensitivity	36 (67)	1.72 (1.78)

**Table 4:** Barriers to consumer shopping behavior.

Barrier type		Participants (N=54), n (%)	Mentions, mean (SD)
Systemic barriers		46 (85)	4.52 (3.9)
**Personal barriers**		54 (100)	10.5 (6.59)
	Attitudes (eg, denial, resignation)	40 (74)	1.74 (1.67)
	Confusion/ignorance	46 (85)	4.44 (4.33)
	Lack of trust	49 (91)	4.31 (3.06)

### Association Between Extent of Positive or Negative Sentiment

The association between extent of positive or negative sentiment expressed by participants and the extent to which each shopping stage was discussed was also examined. Positive sentiment was significantly and positively correlated with Stage 2 in the shopping process (identifying and determining health plan, provider, or treatment options; *r*=0.58; *P*<.001), Stage 4 (making the purchase decision; *r*=0.45; *P*<.001), and Stage 5 (evaluating the purchase decision; *r*=0.38; *P*=.004). Negative sentiment was significantly and positively correlated with Stage 3 (seeking value; *r*=0.30; *P*=.02) and Stage 4 (making the purchase decision; *r*=0.31; *P*=.006).

Finally, we examined the association between positive or negative sentiment and the extent to which each consumer shopping behavior was reported. Positive sentiment was not statistically significantly correlated with any of the behaviors. Negative sentiment was significantly and positively associated with paying for care out-of-pocket or cost sharing (*r*=0.40; *P*<.03), negotiating (*r*=0.45; *P*<.001), and self-advocacy (*r*=0.42; *P*=.001).

## Discussion

### Principal Findings

In this investigation of health consumerism among 54 insured individuals across a range of ages with generally high health care utilization, explicit consumer shopping behaviors—even those not typically associated with health care decision making—were prevalent, though not always successful. Participants perceived pervasive barriers to engaging in health care shopping. Most participants experienced systemic or administrative barriers, and all exhibited personal barriers related to their attitude, knowledge, or trust in the system.

**Figure 2:**
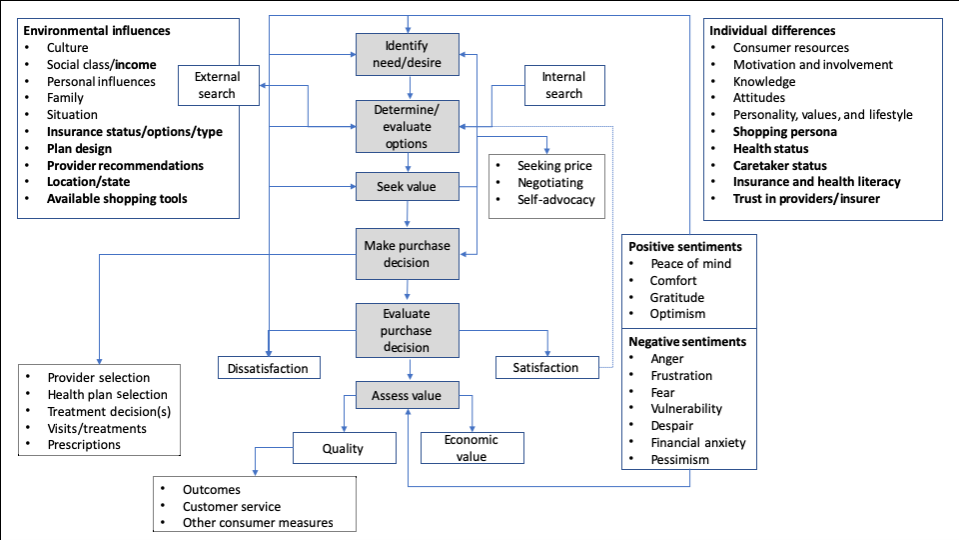
Consumer health care shopping process.

Despite barriers, these results indicate that health care purchasing processes, as different as they may appear from other purchases, could be mapped to established consumer behavior models. We have offered an adaptation of Blackwell/Miniard’s model to account for the findings on the specific nature of and feelings about health care shopping processes [25]. Figure 2 visually depicts findings of the most salient steps and factors in health care purchasing. This model provides a potential alternative to prevailing assumptions that health care purchasing does not reflect traditional consumer shopping behaviors; it also invites further refinement to establish a standard framework for health care shopping processes.

Participants who had purchased individual insurance on either the state or federal health insurance marketplace discussed Stage 2 of the shopping process (determining and evaluating options) more than those with employer-sponsored insurance, suggesting the possible influence of the Affordable Care Act on consumer orientation toward shopping for health insurance.

We found that 98% (53/54) of participants had engaged—or tried to engage—in behaviors relating to seeking, comparing, or knowing prices of care. Specifically, in our study, 72% (39/54) of participants reported seeking information about the cost of care, and 50% (27/54) reported comparing prices or looking for a lower price via an alternate provider. Our study participants had greater prevalence of these behaviors than other studies; Public Agenda [26] found that 50% of participants surveyed had tried to find price information and 20% tried to compare prices between providers. Mehrotra et al [13] found that 13% of subjects had tried to find price information and 3% had tried to compare prices. Both Public Agenda (n=2062) [26] and Mehrotra et al (n=1904) [13] used structured survey instruments with large-scale samples. This study’s smaller sample may be biased toward people with more health care experiences than a general population, and intensive interviews may be more sensitive than survey instruments. Additionally, the open-ended nature of our study may reflect broader interpretation of these behaviors, compared with a close-ended survey question. These results support others’ findings that there is widespread consumer interest in knowing the prices of health care services.

Brand, which is central in many shopping processes and is the focus of billions of health care marketing dollars each year [33], was not a dominant factor in participant provider or health plan selection, with less than a quarter of participants reporting it. This finding may reflect opportunities for more effective branding efforts, or it may reflect the need for health plans and providers to focus on other measures, such as affordability. Finally, it may simply reflect consumers’ own underreporting of the importance of brand as a factor in their selections.

Cost was a consideration for 85% of participants, despite 44% of participants having employer-sponsored insurance, a group with generally lower financial barriers to accessing health care services [33]. This result likely reflects the impact of increased use of high-deductible plans among employers.

The authors also sought to understand how participants feel about health care purchasing by examining how the amount of discussion of a particular shopping stage relates to the amount of positive or negative sentiment expressed by participants. Positive sentiment was significantly and positively correlated with identifying options, making purchase decisions, and evaluating purchase decisions. One previously uninsured participant positively evaluated his individual insurance purchase: “I’m very comfortable with it…I’m pretty pleased to have it.”

Negative sentiment was significantly and positively correlated with seeking value and making the purchase decision. One participant found seeking value following an unsuccessful surgery infuriating:

I have to get revision surgery. If I was not happy with another service, I wouldn’t pay the bill. I would fight the bills. In this case, I have scar tissue that is causing me problems, I still can’t breathe. Why am I still on the hook for a little bit of money?

Positive sentiment associated with identifying options, making decisions, and evaluating decisions may suggest participants’ appreciation for the availability of options and the opportunity to make decisions. Negative sentiment associated with seeking value likely reflects the frustration consumers expressed over systemic barriers to finding cost information and, more generally, to the high cost of health care coverage and services. Negative sentiment associated with making purchase decisions may suggest discomfort among some consumers with available options or a general unease or distaste for needing to function as a health care decision maker.

It cannot be concluded, based on this study design, whether these associations are causal or reflective of more complex relationships. However, these correlations suggest the possibility of interesting relationships between sentiment and consumer shopping processes in health care, which merit further investigation to clarify the nature of the relationships.

The authors also sought to understand how sentiment related to engaging in explicit consumer shopping behaviors and found no relationship between consumer shopping behaviors and positive sentiment. However, negative sentiment was significantly associated with paying out-of-pocket or cost sharing, negotiating, and self-advocacy. As noted, the design of this study does not allow for the determination of causality, but the interviews suggest the relationship may be bidirectional. For example, after a procedure, one participant tried unsuccessfully to negotiate with a doctor who had billed insurance for two separate procedures:

[The doctor] didn’t care, since this is the way they bill it…they expect their money. I paid for that other part, which I didn’t think I should have paid for, and I told them, I said, “You’ve just lost yourself a patient and other references.“

Conversely, another participant’s negative experience led to enhanced consumer behaviors. Undergoing cancer treatment, she experienced a lack of personalization and inadequate access to her providers. These negative experiences led her to more active self-advocacy:

I’m my own advocate. My husband’s my advocate. We are the quarterbacks. . . we learned we had to play [that role]. I did not assume I would need to do this. . . this was my first experience with health care where I realized it’s not up to them, it’s up to me. . .

Further investigation could illuminate the nature of these relationships and the prevalence in a general population.

### Limitations

Multiple limitations in this study should also be noted. First, as a convenience sample, selection bias cannot be excluded in the sample; those who agreed to participate may be more likely to have health care experiences to discuss. Second, the grounded theory method does not search for objective “truth” but rather develops theories based on empirical qualitative data [27,28]. As such, it does not deduce testable hypotheses from existing theories. Critics find grounded theory specifically overly reliant on empirical data, and qualitative methods generally to be anecdotal or impressionistic. However, proponents point to the power of qualitative methods to provide a conceptual understanding of studied phenomena and emergent, original theories [27]. Additionally, temporal, spatial, geographic, and personality or psychological traits or propensity toward positive or negative sentiments may influence participant responses; other than noting optimism or pessimism biases and including those in the sentiment analysis, these factors are not considered [34,35].

Finally, the limited sample size, and particularly small numbers of some subgroups within the sample, may impact the generalizability of our results and preclude additional hypothesis testing.

Nonetheless, these results help to illuminate consumers’ experiences with and attitudes toward health care purchasing. Further examination of the differences by demographic segment and type of purchase (eg, care vs coverage) will advance this effort and determine if these findings apply to different populations. Additionally, large-scale surveys would confirm or refine these findings.

### Conclusions

More generally, these results confirm widely reported obstacles to consumer shopping behaviors in health care, from structural barriers like lack of price transparency to individual constraints like information asymmetry or confusion. On the contrary, these results also reinforce the potential role of market forces in health care and the conceptual relevance of consumer shopping behavior frameworks. Similarly, narrow definitions of consumer-driven health care—as simply high-deductible health plans—ought to be broadened to include a wider range of behaviors and incentives. Such reframing would enable future studies to explore the discordance between consumers’ desire to engage and their ability to do so to capture value in health care purchasing. Recognizing that consumers do shop for health care, and understanding how they shop for health care, are crucial steps in designing interventions to enhance this process.

## Appendix

Multimedia Appendix 1 Frequency of shopping stages.
