# Supplementary material for: Health Care Consumer Shopping Behaviors and Sentiment: Qualitative Study
Source: J Particip Med. 2020 Jun 16;12(2):e13924. doi: 10.2196/13924 (PMC7434061; doi:10.2196/13924)
Supplement: Multimedia Appendix 1 [file jopm_v12i2e13924_app1.docx]

## Multimedia Appendix

Multimedia Appendix 1. Frequency of Shopping Stages

| **Shopping Stage** | **Participants (N=54)** | | **Mentions** | |
| --- | --- | --- | --- | --- |
|  | **n** | **%** | **Mean** | **SD** |
| 1. Identifying the need or desire for a purchase in a health care context | 49 | 91% | 3.98 | 2.61 |
| 1. Determining and evaluating options to meet that need or desire | 54 | 100% | 35.65 | 15.71 |
| 1. Seeking value (a subset of evaluating options) | 54 | 100% | 20.50 | 11.59 |
| 1. Making the purchase decision | 54 | 100% | 15.87 | 8.64 |
| 1. Evaluating the purchase decision in terms of quality and/or satisfaction | 52 | 96% | 7.46 | 4.53 |
| 1. Evaluating value (a subset of evaluating the purchase) | 54 | 100% | 17.74 | 7.78 |
